# Improving Signal-to-Noise Ratio in Susceptibility Weighted Imaging: A Novel Multicomponent Non-Local Approach

**DOI:** 10.1371/journal.pone.0126835

**Published:** 2015-06-01

**Authors:** Pasquale Borrelli, Giuseppe Palma, Enrico Tedeschi, Sirio Cocozza, Marco Comerci, Bruno Alfano, E. Mark Haacke, Marco Salvatore

**Affiliations:** 1 Advanced Biomedical Sciences Department, University of Napoli “Federico II”, Napoli, Italy; 2 Institute of Biostructures and Bioimaging, National Research Council, Napoli, Italy; 3 The MRI Institute for Biomedical Research, Detroit, MI, United States of America; 4 IRCCS SDN, Naples, Italy; The Lee Kong Chian School of Medicine, SINGAPORE

## Abstract

In susceptibility-weighted imaging (SWI), the high resolution required to obtain a proper contrast generation leads to a reduced signal-to-noise ratio (SNR). The application of a denoising filter to produce images with higher SNR and still preserve small structures from excessive blurring is therefore extremely desirable. However, as the distributions of magnitude and phase noise may introduce biases during image restoration, the application of a denoising filter is non-trivial. Taking advantage of the potential multispectral nature of MR images, a multicomponent approach using a Non-Local Means (MNLM) denoising filter may perform better than a component-by-component image restoration method. Here we present a new MNLM-based method (Multicomponent-Imaginary-Real-SWI, hereafter MIR-SWI) to produce SWI images with high SNR and improved conspicuity. Both qualitative and quantitative comparisons of MIR-SWI with the original SWI scheme and previously proposed SWI restoring pipelines showed that MIR-SWI fared consistently better than the other approaches. Noise removal with MIR-SWI also provided improvement in contrast-to-noise ratio (CNR) and vessel conspicuity at higher factors of phase mask multiplications than the one suggested in the literature for SWI vessel imaging. We conclude that a proper handling of noise in the complex MR dataset may lead to improved image quality for SWI data.

## Introduction

Susceptibility-weighted imaging (SWI) is based on the differences in tissue susceptibility to enhance the contrast in magnitude MR images [1]. In this technique, the phase from local field inhomogeneities is used as the source of contrast in order to reveal important anatomical and physiological information about vessels and tissues containing iron [2–4]. In particular, the merged magnitude/phase SWI data offers information about any tissue that has a different susceptibility than surrounding tissues and is particularly useful in reflecting the presence of deoxygenated blood, ferritin and hemosiderin. Although used mainly in neuroimaging to study the intracranial venous system [5–9], SWI images have recently been applied in other parts of body as well [10–12]. However, the high resolution required to obtain sufficient phase information, which can be used for improved contrast, may lead to a reduced signal-to-noise ratio (SNR), thus compromising both postprocessing tasks and the overall visual inspection.

In the context of denoising algorithms, the non-local means (NLM) filter [13] has proven to be one of the best performing and robust denoising methods. The NLM filter is a non-linear neighborhood filter in which the voxel value to be restored is replaced by a weighted average of the voxel intensities in the entire noisy image. Unlike other neighborhood filters, the weights are determined by the neighborhood similarity on the basis of the intensities in patches surrounding each voxel of the image. In other words, the NLM algorithm can be seen as a patch-based mean filter in which the similarity between patches surrounding each voxel is used in the restoring process rather than the intensities of the voxel themselves. Furthermore, the region comparison is based on the radiometric proximity instead of geometrical distance between patches, therefore making it non-local. It has been found that the NLM filter outperforms other existing denoising schemes and, in particular, it is efficient when applied to MR datasets [14–16]. Moreover, to denoise multichannel images, the redundant information between different components is used in the non-local metric as a way to better distinguish the noise from image features. Taking into account the multispectral nature of MR data, Manjòn et al. [17] defined a multicomponent approach (hereafter MNLM) as a basis for the denoising process. Recent studies [18] take into account the spatially varying noise distribution of MR data acquired with parallel imaging techniques, thus avoiding the introduction of bias due to non-uniform noise distribution in parallel acquisitions.

To the best of our knowledge, the noise removal algorithm in the SWI pipeline has had limited investigation. In a recent study, noise removal in SWI images was performed using a NLM version in the complex domain, the so-called Imaginary-Real-SWI (IR-SWI, [19]). Even though the IR-SWI scheme obtained an improvement in image quality, it did not take into account the multicomponent property of the SWI images, thus producing blurred images. Here we present a new method (Multicomponent-Imaginary-Real-SWI, hereafter MIR-SWI) based on the MNLM denoising algorithm, to produce SWI images with both high SNR and improved conspicuity.

The plan of the paper is as follows. In §1 we briefly review the standard SWI generation pipeline (§1.1), the NLM denoising algorithm (§1.2) and the noise estimation in MR images (§1.3). Then, in §2 we describe the acquisition protocol (§2.1) and present the details of the NLM schemes we tested in order to denoise magnitude and phase data to produce the restored SWI images (§2.2). Finally, in §3 and §4 we present and discuss the results, showing the performance of our algorithm in comparison with other state-of-art denoising schemes.

## Analysis

### 1.1 SWI magnitude image

In order to generate susceptibility-weighted magnitude images, the information from phase data is used to create a mask that enhances local changes in tissue magnetic susceptibility. As reported in [1], a high-pass filter (*hp*) is applied to phase image (𝜑) to obtain *𝜑_hp_* and remove low spatial frequency contributions from field variations due to external field inhomogeneities. Then, the SWI image is computed as follows:

$$\begin{array}{c} SWI\left(I_{m},\Phi_{hp}\right)=I_{m}\cdot \Phi_{mask}^{n}\left(\Phi_{hp}\right), \end{array}$$ (1)

where *I_m_* is the magnitude image,

$$\begin{array}{c} \Phi_{mask}\left(\Phi_{hp}\right)=\{\begin{array}{cc} 1-\frac{|\Phi_{hp}|}{\pi }, & \text{if}-\pi <\Phi_{hp}<0,\\ 1, & \text{else}. \end{array} \end{array}$$ (2)

(for a right-handed system 𝜟𝜑< 0 corresponds to a paramagnetic behavior) and *n* is a parameter (usually set to 4) to tune in order to optimize the contrast-to-noise ratio (CNR) in the SWI image.

### 1.2 Non-local means algorithm

An *N*-D image *X* can be considered as a real function *X* : ℝ^*N*^ → ℝ with a bounded support Ω ⊂ ℝ^*N*^. The NLM filter [13] is a class of endomorphisms of the image space, identified by 2 parameters (*a* and *h*), that acts as follows:

$$\begin{array}{c} \left[{\mathrm{NLM}}_{a,h}\left(X\right)\right]\left(\vec{x}\right)=Y\left(\vec{x}\right)=\frac{\int_{\Omega }\exp[-\frac{d_{a}^{2}\left(\vec{x},\vec{y}\right)}{h^{2}}]X\left(\vec{y}\right)d\vec{y}}{\int_{\Omega }\exp[-\frac{d_{a}^{2}\left(\vec{x},\vec{y}\right)}{h^{2}}]d\vec{y}}\;, \end{array}$$ (3)

where

$$\begin{array}{c} d_{a}^{2}\left(\vec{x},\vec{y}\right)\equiv \int_{{\mathbb{R}}^{N}}{\left|X\left(\vec{x}+\vec{t}\right)-X\left(\vec{y}+\vec{t}\right)\right|}^{2}\cdot \frac{\exp-\frac{{∥\vec{t}∥}^{2}}{2a^{2}}}{{\left(2\pi \right)}^{n/2}\cdot a}d\vec{t}\;, \end{array}$$ (4)

*a* represents the radius of the window centered on each point of the image and *h* rules the similarity measure in the window comparison.

Both computational issues and the convenience to introduce a geometric proximity criterion in addition to the pure radiometric distance measure led to a change in the original version of the NLM filter [15]. More specifically, given a search radius *M*, for each voxel *i* located at ${\vec{x}}_{i}$ we define a search box *V_i_* as

$$\begin{array}{c} V_{i}\equiv \{{\vec{x}}_{j}\in \Omega |{∥{\vec{x}}_{j}-\vec{x_{i}}∥}_{\infty }<M\}\;. \end{array}$$ (5)

The search box *V_i_* defines the ensemble of voxels whose intensities will be available to restore the voxel located in ${\vec{x}}_{i}$, thus reducing the search freedom.

Likewise, given a similarity radius *d* ∼ *a*, for each voxel ${\vec{x}}_{j}$ within a given search box *V_i_*, we can define a similarity box *_j_B_i_* as

$$\begin{array}{c} {}_{j}B_{i}\equiv \{{\vec{x}}_{k}\in \Omega |{∥{\vec{x}}_{k}-\vec{x_{j}}∥}_{\infty }<d\}\;. \end{array}$$ (6)

If the image is defined on a discrete grid, a suitable filter implementation is:

$$\begin{array}{c} Y_{i}=\frac{\sum_{{\vec{x}}_{j}\in V_{i}}\exp[-\frac{{∥{}_{j}B_{i}-{}_{i}B_{i}∥}_{2}^{2}}{h^{2}}]X_{j}}{\sum_{{\vec{x}}_{j}\in V_{i}}\exp[-\frac{{∥{}_{j}B_{i}-{}_{i}B_{i}∥}_{2}^{2}}{h^{2}}]}\;, \end{array}$$ (7)

The filter strength, which is determined by *h*, can be automatically tuned to obtain an optimized denoising, independent of the search radius *M* and the standard deviation of noise *σ*:

$$\begin{array}{c} h^{2}=2\beta \sigma^{2}|V_{i}| \end{array}$$ (8)

(*β* ∼ 1 is an adimensional constant to be manually tuned).

For a multispectral framework approach, the filtering process can be improved by using intercomponent information to discriminate between noise and image features and reveal masked image details or discard false structures generated by noise. In this setting, the similarity measure in the NLM algorithm can be improved by combining not only the information of surrounding voxels within the image but also the information of different components in order to take advantage of the redundancy between MR series. According to [17], for each component *c* the filter implementation in Eq (7) is extended to be used on a multispectral framework as follow:

$$\begin{array}{c} Y_{i,c}=\frac{\sum_{{\vec{x}}_{j}\in V_{i}}\exp\left[-\frac{1}{C}\sum_{c=1}^{C}\frac{{∥}_{j}B_{i,c}{-}_{i}B_{i,c}{∥}_{2}^{2}}{2\beta \sigma_{c}^{2}\left|V_{i,c}\right|}\right]X_{j,c}}{\sum_{{\vec{x}}_{j}\in V_{i}}\exp\left[-\frac{1}{C}\sum_{c=1}^{C}\frac{{∥}_{j}B_{i,c}{-}_{i}B_{i,c}{∥}_{2}^{2}}{2\beta \sigma_{c}^{2}\left|V_{i,c}\right|}\right]}, \end{array}$$ (9)

where *C* represents the number of components and *σ*_*c*_ is the standard deviation of each component.

### 1.3 Noise estimation in MR images

Eq (8) shows that noise standard deviation of the MR images must be estimated in order to make the smoothing strength of the MNLM filter independent of the noise power. In the context of MRI, the real and imaginary components of the complex acquired signal are assumed to be corrupted with independent and identically distributed Gaussian noise. Due to the non-linear transformation leading to magnitude and phase images, the noise distribution is no longer Gaussian [20]. Moreover, for parallel imaging, the application of multi-surface coil arrays and reconstruction filter can influence the statistical distribution of image noise [21]. As reported in [18], to overcome the different noise distribution between magnitude and phase data and to avoid introducing a bias due to spatially varying noise properties in parallel MR acquisitions, noise standard deviation is evaluated by a local statistic method. In this setting, *σ* in Eq (8) represents a function of local noise amplitude.

## Materials and methods

### 2.1 Acquisition protocol

Two axially-oriented fully flow-compensated spoiled gradient echo sequences were acquired on a Siemens Trio 3T scanner in 4 healthy volunteers using an 8-channel head receiver coil. The “Carlo Romano” ethics committee for biomedical activities of “Federico II” University of Naples (Italy) specifically approved the study and the written informed consent form, which was signed by all subjects undergoing the MR scan. Common acquisition parameters included a flip angle of 15 degrees, repetition time of 28 ms, echo time of 22.14 ms (in phase water and fat component), field of view of 230 × 194 × 166 mm^3^, acquisition matrix of 320 × 270 × 128, a GRAPPA factor of 2 and an acquisition time of 5 min ans 8 s. The two acquisitions differed from each other in read-out bandwidth only, being 100 and 600 Hz/pixel, respectively. Unfiltered magnitude and phase reconstruction was enabled, thus obtaining a complex volume dataset for each acquisition.

Since in MRI the SNR is related to the square root of the bandwidth, we selected 100 Hz for a high SNR acquisition (kind of “reference acquisition”). In fact, that value is indeed close to the lowest bandwidth limit compatible with clinical research protocols using SWI, as the echo time should not be increased beyond the values suggested in the literature [22] to keep the contrast unchanged.

On the other hand, using the higher bandwidth of 600 Hz/pixel (“noisy acquisition”) yields a 2.5-fold decrease in SNR, but does not affect contrast in the image.

### 2.2 SWI Denoising

In this section we describe several pipeline configurations tested in order to investigate the performance of currently applied denoising schemes in SWI image generation. Due to the high computational complexity of the employed denoising schemes, we used a multi-GPU implementation [23] of the MNLM filter.

**Preliminary investigation** Four different pipeline configurations were implemented over and above the proposed MIR-SWI approach.

For the first implementation, the standard NLM filter was applied downstream of the SWI image generation (hereafter as NLM-SWI).

Second, we processed the complex dataset according to IR-SWI denoising scheme described in [19].

For the next test, taking into account the multispectral nature of MR series, we applied the MNLM algorithm on magnitude and phase images before SWI generation (hereafter indicated as MNLM-SWI). In more detail, *φ* and *I_m_* images were first restored with MNLM algorithm and subsequently the MNLM-SWI image was computed according to Eq (2) and Eq (1). Since the phase image is limited to the domain [-*π*, +*π*), in the MNLM-SWI pipeline an unwrapping method must be used before the application of the MNLM denoising filter to recover the true phase on missing multiples of 2*π*. In that scheme, phase images were unwrapped according to [24].

As fourth test (referred to as MNLM-HP-SWI), we revisited more deeply the SWI pipeline. In order to produce the restored SWI image, the MNLM filter was applied to magnitude and high-pass filtered phase images immediately before the phase mask generation, obtaining ${\hat{I}}_{m}$ and ${\hat{\varphi }}_{hp}$. The MNLM-HP-SWI image was then obtained applying Eq (1) on ${\hat{I}}_{m}$ and ${\hat{\varphi }}_{hp}$.

**MIR-SWI method** The proposed MIR-SWI scheme consists of a complex domain-based application of the MNLM filter. Since the full k-space acquired in MRI is assumed to be corrupted with Gaussian white noise, after Fourier transform real and imaginary images are still corrupted by uncorrelated Gaussian noise with the same variance in both complex components [20]. In this setting, we removed unwanted low-frequency *B_0_* variation by defining real and imaginary images as:

$$\begin{array}{c} I_{R}=I_{m}\cdot \cos\left(\Phi_{hp}\right) \end{array}$$ (10)

$$\begin{array}{c} I_{I}=I_{m}\cdot \sin\left(\Phi_{hp}\right) \end{array}$$ (11)

and applied MNLM algorithm to *I_R_* and *I_I_*.

After denoising ${\tilde{I}}_{R}$ and ${\tilde{I}}_{I}$, the restored magnitude (${\tilde{I}}_{m}$) and phase (${\tilde{\varphi }}_{hp}$) images are derived as

$$\begin{array}{c} {\tilde{I}}_{m}=\sqrt{{\tilde{I}}_{R}^{2}+{\tilde{I}}_{I}^{2}}\;, \end{array}$$ (12)

$$\begin{array}{c} {\tilde{\Phi }}_{hp}=∠\left({\tilde{I}}_{R}+i{\tilde{I}}_{I}\right)\;, \end{array}$$ (13)

and then processed according to Eq (1).

## Results

**Fig 1:**
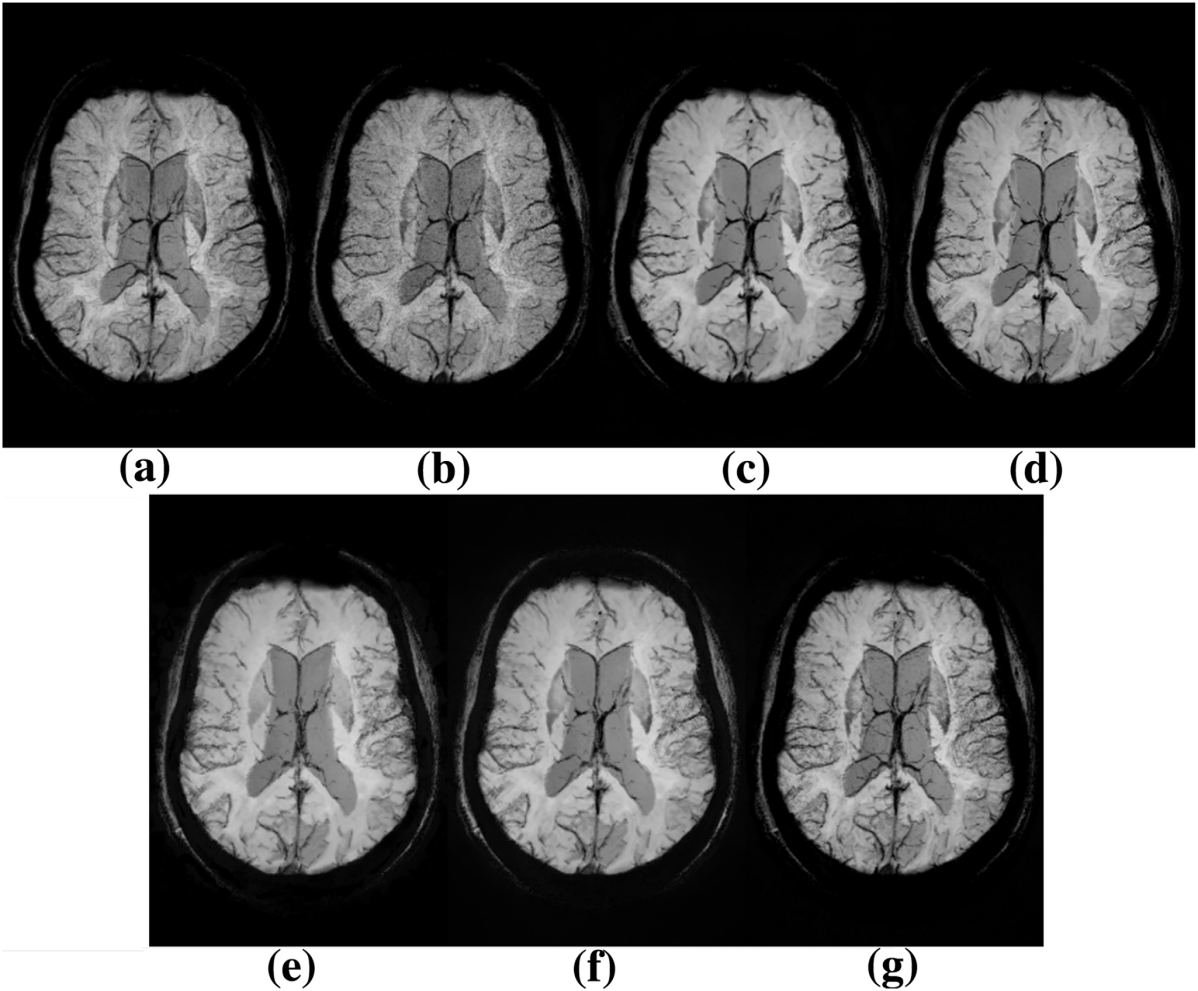
Results of different denoising pipelines on SWI image generation. Axial brain mIPs (corresponding to a volume of 20 mm) at the level of the lateral ventricles of SWI-100Hz (a), SWI (b), NLM-SWI (c), IR-SWI (d), MNLM-SWI (e), MNLM-HP-SWI (f), and MIR-SWI (g) images. The number of phase mask multiplications is set to 4. Enhanced visibility of venous structures without loss of tissue contrast is evident in (g) compared to (b-f).

**Fig 2:**
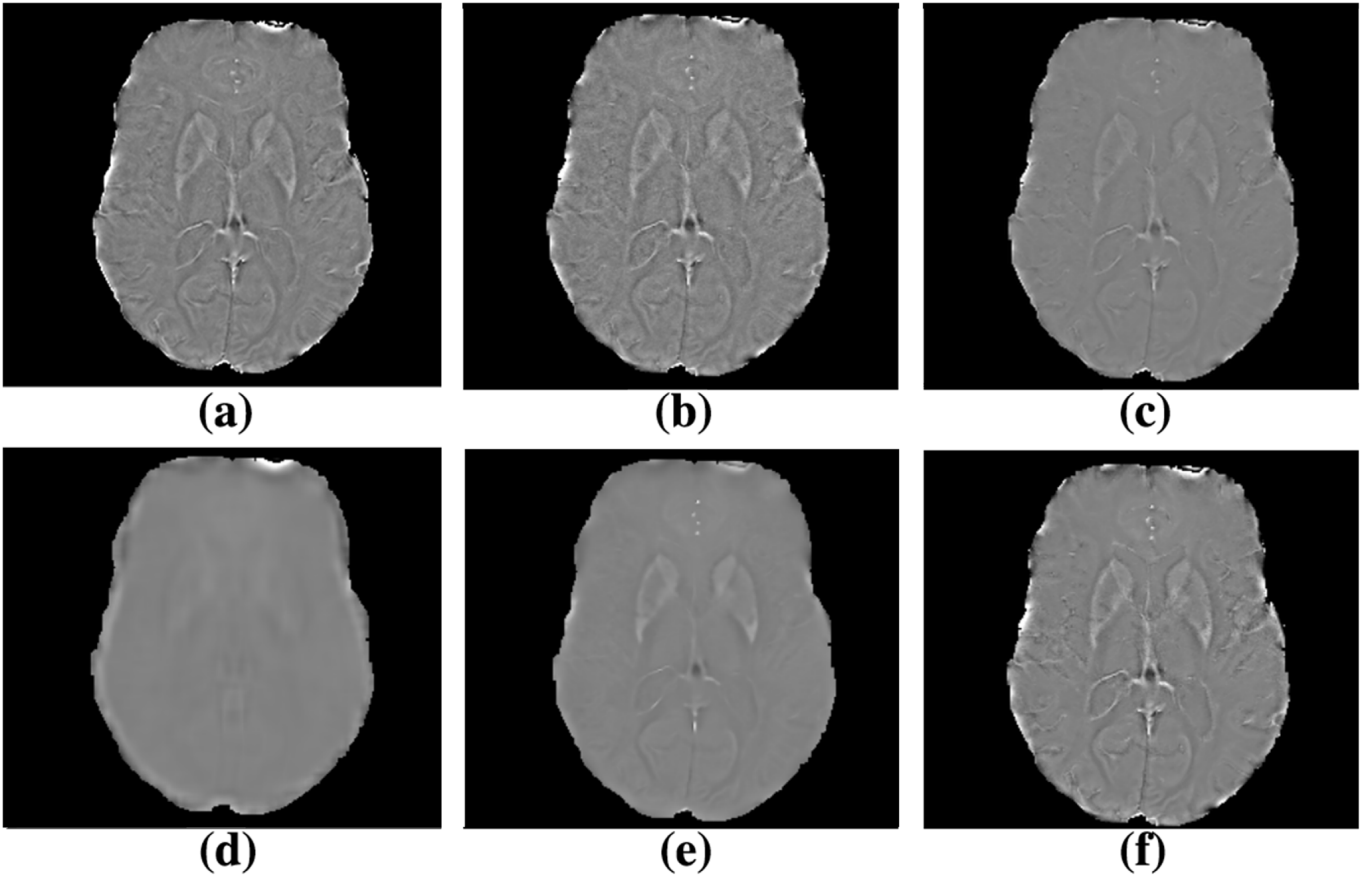
Influence of different denoising pipelines on high-pass filtered phase images. Argument of the phase mask function (somehow equivalent to high-pass filtered phase) in the following pipelines: SWI-100Hz (a), SWI (b), IR-SWI (c), MNLM-SWI (d), MNLM-HP-SWI (e) and MIR-SWI (f). The tissues outside the brain were masked in order to focus on the denoising results. The image obtained with MIR-SWI scheme shows good noise suppression while preserving brain structures compared to both MNLM-SWI and MNLM-HP-SWI images.

In Fig 1 we summarize the denoising outcomes obtained by testing the different NLM pipelines described in §2.2 compared to the proposed method. In Fig 2 we show the denoising results on high-pass filtered phase images before the SWI image generation. The denoising performance of the proposed scheme was compared to the original SWI, to the NLM-SWI, to the MNLM-SWI, to MNLM-HP-SWI and to IR-SWI. To assess both the correct noise removal and the preservation of edges and tiny structures, we used a high-SNR SWI dataset acquired with a bandwidth of 100 Hz/pixel (labelled as SWI-100Hz) as the “reference SWI”.

### Qualitative assessment (brain tissues)

In order to evaluate the performance of MIR-SWI in comparison with original SWI, NLM-SWI, MNLM-SWI, MNLM-HP-SWI and the IR-SWI, the six sets of images from 4 healthy volunteers were randomly presented to two neuroradiologists with more than 20-years experience in MR neuroimaging as 50 sextets of corresponding axial slices at different brain levels from the foramen magnum to the vertex. Semiquantitative assessment of the images was performed blindly and in consensus by rating the gray/white matter differentiation, the presence of artifacts at the brain/cerebrospinal fluid interface, or any other obvious artifacts, the confidence in detecting clinical relevant findings and the overall image quality, on a 0–5 scale, with 5 being the best representation of intracranial structures without relevant artifacts and 0 the worst and clinically inadequate display.

**Fig 3:**
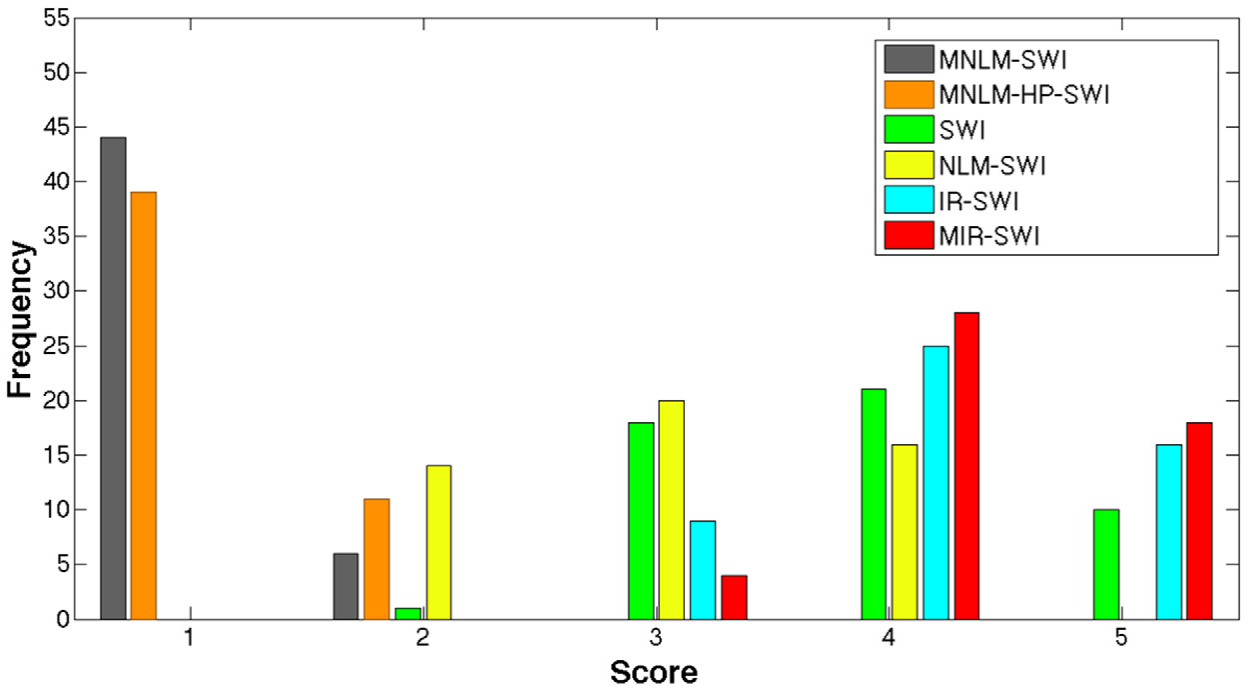
Semiquantitative visual assessment. Frequency histogram of the semiquantitative scores for the display of the brain structures of the MNLM-SWI (gray), MNLM-HP-SWI (orange), SWI (green), NLM-SWI (yellow), IR-SWI (cyan) and MIR-SWI (red) images. Score values from 1 to 5 indicate increasing overall image quality (see text).

The MIR-SWI images scored 4 or 5 in 46 cases (92%), the SWI images scored 4 or 5 in 31 cases (62%), the IR-SWI images scored 4 or 5 in 41 cases (82%) while standard NLM-SWI scored 4 or 5 in only 16 cases (32%), and were never preferred over the corresponding SWI counterpart (Fig 3).

MNLM-SWI and MNLM-HP-SWI schemes clearly produced images of systematically lower quality (e.g., sharpness, vein conspicuity, gray/white matter differentiation), as shown in Fig 1 and in Fig 2 and confirmed by the semi-quantitative analysis in Fig 3. For this reason, the quality metrics computed in the subsequent analysis for these two schemes are not shown.

### Quantitative evaluation (veins)

The denoising performance was quantitatively evaluated both by a vein-based contrast-to-noise ratio (VB-CNR) comparison and by a vessel-profile analysis.

**Fig 4:**
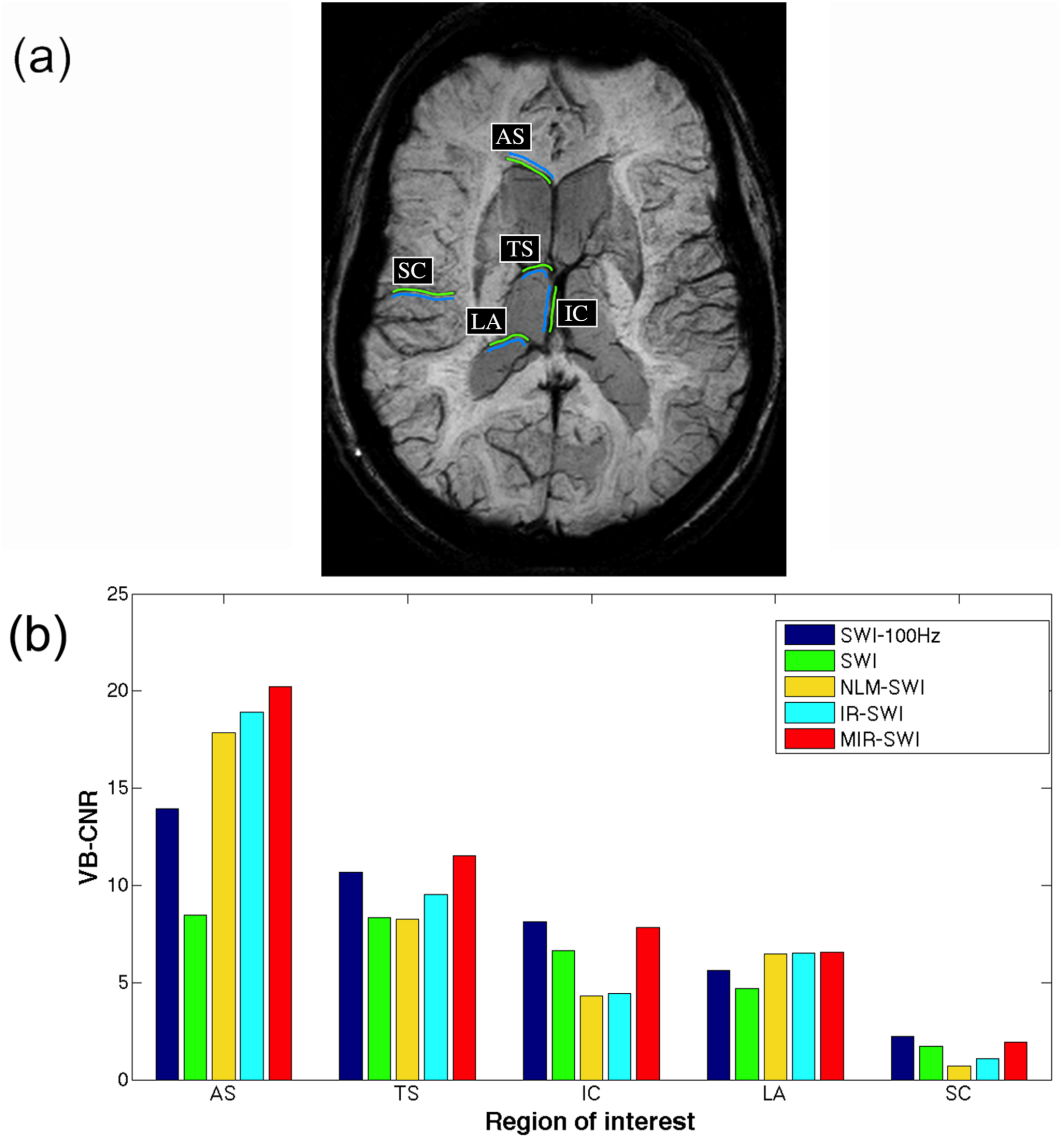
VB-CNR analysis in subject #1. SWI-100HZ axial brain mIP (a) corresponding to a volume of 20 mm shows the five venous ROIs we used for the quantitative evaluation of the MIR-SWI denoising scheme. Green lines represent the veins used for VB-CNR analysis while cyan lines are the background counterparts positioned on neighbooring tissues (anterior septal vein, AS; thalamostriate vein, TS; internal celebral vein, IC; lateral atrial vein, LA; silvian cortical vein, SC). The VB-CNR bar graph of each vein (b) shows an overall higher contrast between veins and background of the MIR-SWI (red bars) compared to the other schemes.

As proposed by Jang et al. [25], VB-CNR was defined as:

$$\begin{array}{c} VB-CNR_{ab}=\frac{\left|S_{a}-S_{b}\right|}{\sigma_{b}}\;. \end{array}$$ (14)

where *S_a_* and *S_a_* are the mean signal intensities in region *a* and *b* and *σ*_*b*_ is standard deviation of region *b*. Region *a* is defined as the set of pixels on a line passing through the center of the vein whereas region *b* is the set of pixels on a line surrounding the vein [25]. Using the same approach, in the present study a single observer manually segmented five veins: anterior septal vein (AS), thalamostriate vein (TS), internal cerebral vein (IC), lateral atrial vein (LA) and a silvian cortical vein (SC), and measured their VB-CNRs (Fig 4a). In Fig 4b the VB-CNR values for each SWI scheme (SWI, NLM-SWI, IR-SWI and MIR-SWI) and for each vein are compared in a healthy subject (a similar display is reported for the remaining 3 subjects in S4 Fig–S6 Fig).

**Fig 5:**
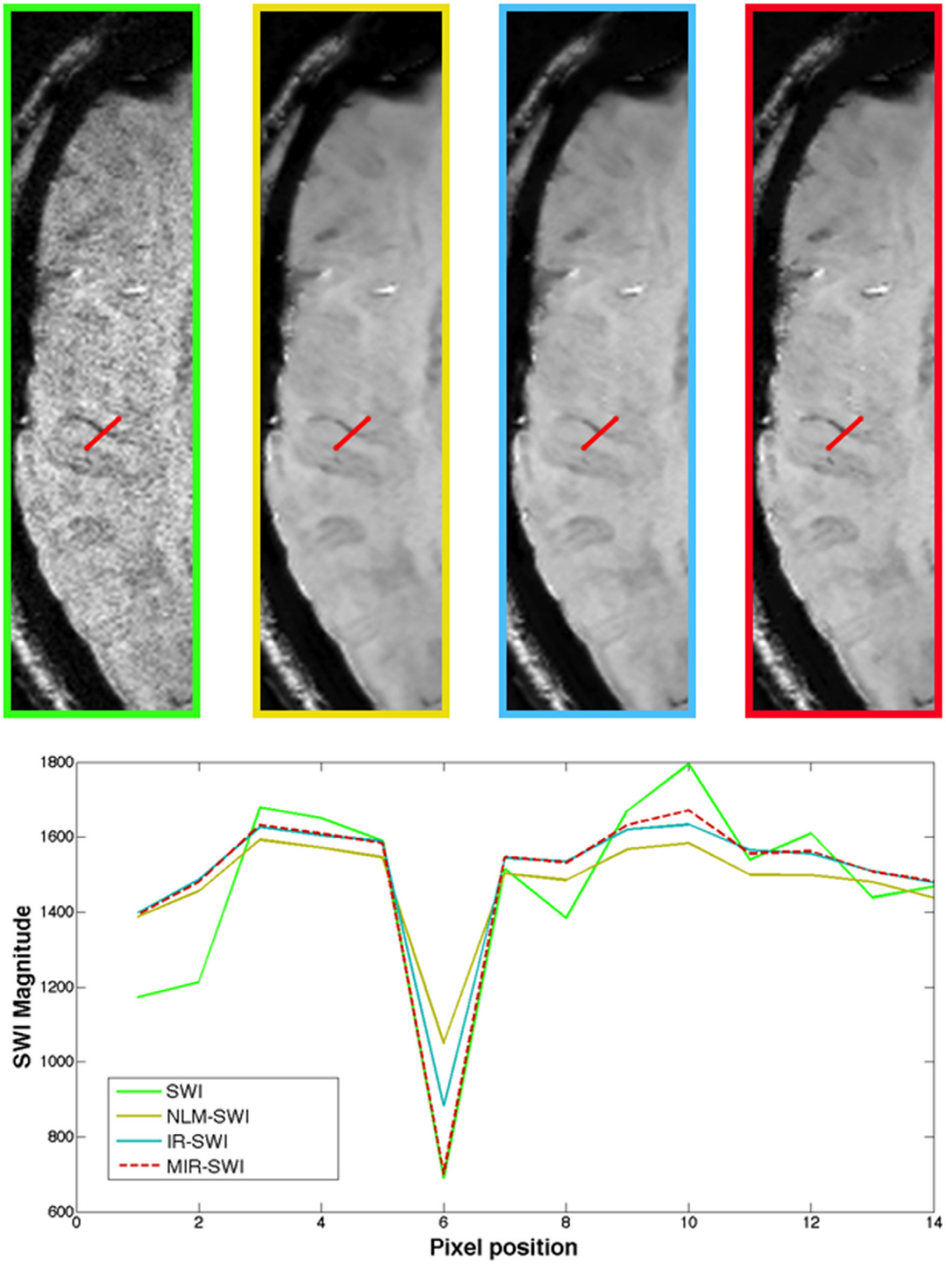
Vessel-profile comparison. Top: SWI, NLM-SWI, IR-SWI and MIR-SWI axial brain slices (from left to right respectively) in a healthy volunteer. The red lines represent the domain used to plot the in-plane profiles of the voxel intensities perpendicular to a small right frontal vein. Bottom: the comparison of the corresponding in-plane profiles of the SWI (green line), NLM-SWI (yellow line), IR-SWI (cyan line) and MIR-SWI (dotted red line) voxel intensities shows that MIR-SWI, IR-SWI and NLM-SWI schemes enhance the SNR of the parenchyma (depicted by the line plateau) compared to the SWI vessel profile, but only the MIR-SWI does not introduce a detrimental blurring between the vessel and surrounding tissues.

Another quantitative description of the denoising effects on the contrast changes between tissues and an estimate of possible edge blurring is given by vessel-profile analysis [26]. As an example, in Fig 5 we report the plotting of in-plane profiles of SWI, NLM-SWI, IR-SWI and MIR-SWI voxel intensities perpendicular to a cortical small vein.

### Number of phase mask multiplications

**Fig 6:**
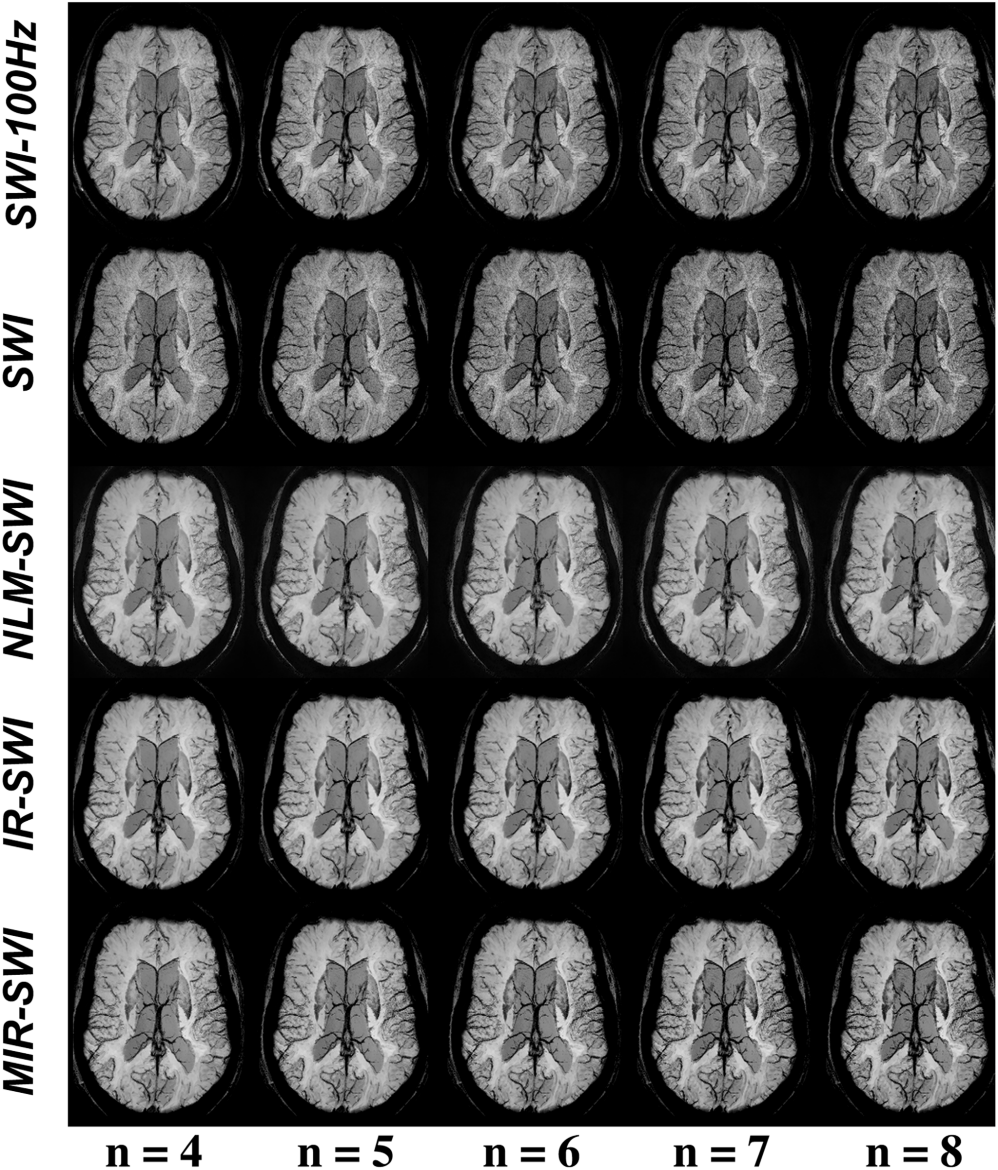
The effect of the ***n*** values on SWI images in subject #1. mIPs of the same targeted volume of 20 mm at varying *n* values. In reference to the SWI-100Hz image, MIR-SWI shows both satisfactory noise removal and better vessel enhancement at increasing *n* values compared to the other SWI schemes.

**Fig 7:**
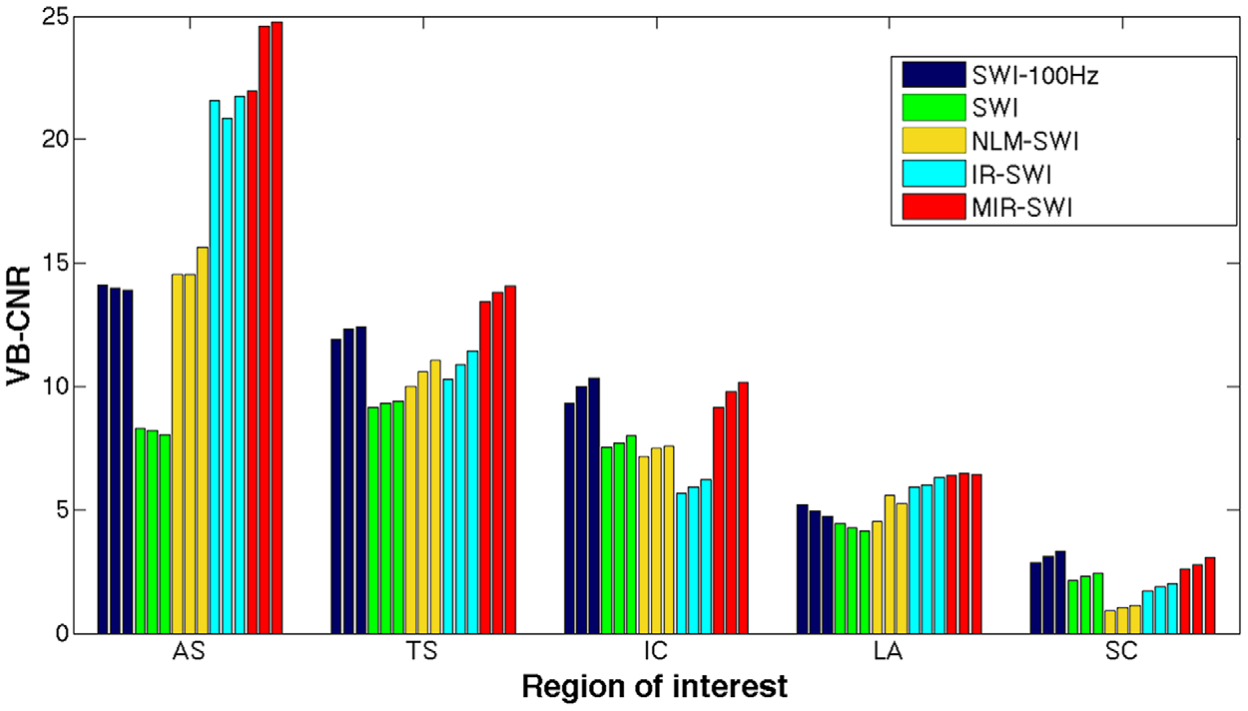
Bar graph of VB-CNRs at different *n* values in subject #1. VB-CNR analysis performed on the same veins of Fig 4a (AS: anterior septal vein, TS: thalamostriate vein, IC: internal cerebral vein, LA: lateral atrial vein, SC: sylvian cortical vein) as they appeared in the three rightmost columns of Fig 6. From each vein, the triplets of bars with the same color correspond to the images with *n* value of 6, 7 and 8, from left to right, respectively. Among the denoising schemes under evaluation, only MIR-SWI (red bars) consistently showed increased VB-CNR in all selected veins.

From Eq (1) one sees that the number of phase mask multiplications may be optimized in order to enhance the contrast between veins or gray matter versus the surrounding tissues while keeping the noise level within reasonable limits. In this setting, we tested different values of phase mask multiplications both in terms of visual inspection (Fig 6, S1 Fig–S3 Fig) and of VB-CNR (Fig 7, S7 Fig–S9 Fig).

## Discussion

To the best of our knowledge, noise removal algorithms in a SWI pipeline have received little attention to data, perhaps even the application of a robust denoising filter as the NLM algorithm produces poor results on SWI images. The excessive blurring and loss of anatomical structures shown in Fig 1c is likely to be caused by an incomplete exploitation of the information contained within the two complex channels. In this context, the generation of a robust method to both enhance the vessels and increase the CNR of SWI images is a non-trivial operation.

A new strategy to restore SWI images based on a dedicated NLM scheme has been recently developed [19]. In that approach, the restoring process of SWI images was solved by applying the NLM filter on the complex image domain between the high-pass filtering step and the phase mask generation, thus taking advantage of the uncorrelated Gaussian distribution with the same noise variance in the real and imaginary parts of the complex image. In that study, while effective noise removal led to an evident noise reduction and a SNR increase, the restoring pipeline did not take into account the multispectral nature of the SWI scheme, thus producing blurred images with reduced differentiation between the brain regions and loss of tiny structures such as small vessels (Fig 1d).

In this article, a new denoising pipeline based on a NLM algorithm to restore SWI images is presented. We tested several pipeline configurations of the multispectral version of the NLM denoising filter [17]. As we outline in Fig 2d, due to incorrect noise removal in phase image, we obtained lacking phase contribution in SWI image when MNLM-SWI method was applied, resulting in an extensive smoothing and loss of phase contrast (Fig 1e). Even the MNLM-HP-SWI scheme similarly produces unsatisfactory outcomes: in fact although the phase information in the MNLM-HP-SWI scheme (Fig 2e) is higher compared to the phase image processed with the MNLM-SWI method, we obtained poor anatomical information due to the different noise distribution between magnitude and high-pass filtered phase images. As outlined in Fig 1f, the contrast in SWI images is generated by an additional phase contribution, but the contrast from some small vessels is missing.

The key in our MIR-SWI approach is to revisit the SWI pipeline by applying a MNLM filter on real and imaginary components of computed complex data between the phase high-pass filtering stage and the phase mask evaluation. The main benefit of the proposed method is to reduce the noise propagation in the non-linear SWI pipeline, thus avoiding the introduction of the biases due to a non-null first moment of the transformed zero-mean white Gaussian noise in complex images. Moreover, the noise removal in the phase image is addressed by considering also the magnitude information into the weighted averaging process, thus taking advantage of the multispectral property of the SWI scheme.

Our results demonstrate that the proposed method clearly improves the SNR and properly preserves the brain structures (Figs 1g–2f). In fact, the visual assessment by two neuroradiologists showed that MIR-SWI images consistently displayed better gray/white matter differentiation, fewer artifacts and improved image quality (Fig 3). Moreover, the visibility of vessels is enhanced due to an increased CNR between the vessels and the surrounding tissues (Figs 4b–5). Unlike NLM-SWI, MIR-SWI images show a better preservation of faint structures revealed by the SWI phase mask, still guaranteeing a high SNR. Finally, compared to IR-SWI images, we obtained both better vein visibility and increased contrast between brain structures when MIR-SWI is applied, thus producing a benefit for quantitative techniques that rely on good quality of the data (such as the quantitation of brain iron content).

Taking into account the noise propagation during the SWI image generation, Haacke et al. [1] proposed to set the number of phase mask multiplications to 4, in order to obtain the best compromise between vessel enhancement and overall image quality. In this setting, we tested several phase mask multiplication values to achieve the most satisfactory phase contrast in SWI images. As pointed out in §3, with the proposed method we can obtain better phase contribution at higher *n* levels without a significant increase of noise, thus resulting in a clear improvement in both CNR and vessel visibility. This is particularly true when the minimum Intensity Projection (mIP) is performed (Fig 6), a reformat technique in which noise propagation is more evident. Beside being preferable in the qualitative assessment of SWI images at different *n* values processed with the SWI schemes under evaluation (Fig 6), MIR-SWI quantitatively showed increased VB-CNR in all selected veins (Fig 7). Although the optimal choice of the *n* value was not the primary aim of this work, we demonstrated that the proper handling of the noise in the MIR-SWI scheme may be used to increase the contrast with different *n* values. In fact, only MIR-SWI guarantees a positive trend in CNR in every vein within the considered range of *n* values. Moreover, MIR-SWI, compared to SWI-100Hz, proved capable of largely filling a 6-fold gap between the two acquisition bandwidths, as shown in Fig 7 where red bars were never shorter than the corresponding blue bars.

VB-CNR values could not be reasonably compared across different subjects, due to marked individual differences in vessel features (e.g., vein caliber, ferromagnetic load, haemodynamic parameters, etc.), that prevent a proper reproducibility analysis. However, the robustness of MIR-SWI can be inferred by evaluating the VB-CNR values of the different veins within the same subject, due to known intra-subject vessel variability. As shown in Figs 4 and 7, VB-CNR values of different veins can be considered representative of the performance variability of the different SWI schemes under a wide range of analyzed subjects. As an additional proof, a Supporting Information file has been added, showing the images of the 3 remaining studies processed with the different SWI pipelines under testing (S1 Fig–S3 Fig) and the corresponding bar graphs of the VB-CNRs (S4 Fig—S9 Fig).

MIR-SWI takes advantage of a multi-GPU implementation of a generic NLM-scheme (see [23]), and is computationally feasible within clinically acceptable times (∼ 3 minutes for a 3D 320 × 270 × 128 complex dataset with a typical denoising parameter setup [*d* = 1, *M* = 5] and 2 NVIDIA GeForce GTX 690).

In summary, our findings may prompt the application of the proposed algorithm to SWI processing in larger patient databases to assess whether clinical usefulness may be improved. Moreover, it may be used to enhance the overall quality of finer phase-image processing, such as Susceptibility Weighted Image Mapping (SWIM) or Quantitative Susceptibility Mapping (QSM) [27].

## Supporting Information

S1 Fig The effect of the *n* values on SWI images. mIPs of the same targeted volume of 20 mm at varying *n* values. Subject #2.
(TIF)

S2 Fig The effect of the *n* values on SWI images. mIPs of the same targeted volume of 20 mm at varying *n* values. Subject #3.
(TIF)

S3 Fig The effect of the *n* values on SWI images. mIPs of the same targeted volume of 20 mm at varying *n* values. Subject #4.
(TIF)

S4 Fig VB-CNR analysis in subject #2. Same legend as Fig 4b.
(TIF)

S5 Fig VB-CNR analysis in subject #3. Same legend as Fig 4b.
(TIF)

S6 Fig VB-CNR analysis in subject #4. Same legend as Fig 4b.
(TIF)

S7 Fig Bar graph of VB-CNRs at different *n* values in subject #2. Same legend as Fig 7.
(TIF)

S8 Fig Bar graph of VB-CNRs at different *n* values in subject #3. Same legend as Fig 7.
(TIF)

S9 Fig Bar graph of VB-CNRs at different *n* values in subject #4. Same legend as Fig 7.
(TIF)
