# Supplementary figures and images for: Improving Signal-to-Noise Ratio in Susceptibility Weighted Imaging: A Novel Multicomponent Non-Local Approach
Source: PLoS One. 2015 Jun 1;10(6):e0126835. doi: 10.1371/journal.pone.0126835 (PMC4452483; doi:10.1371/journal.pone.0126835)

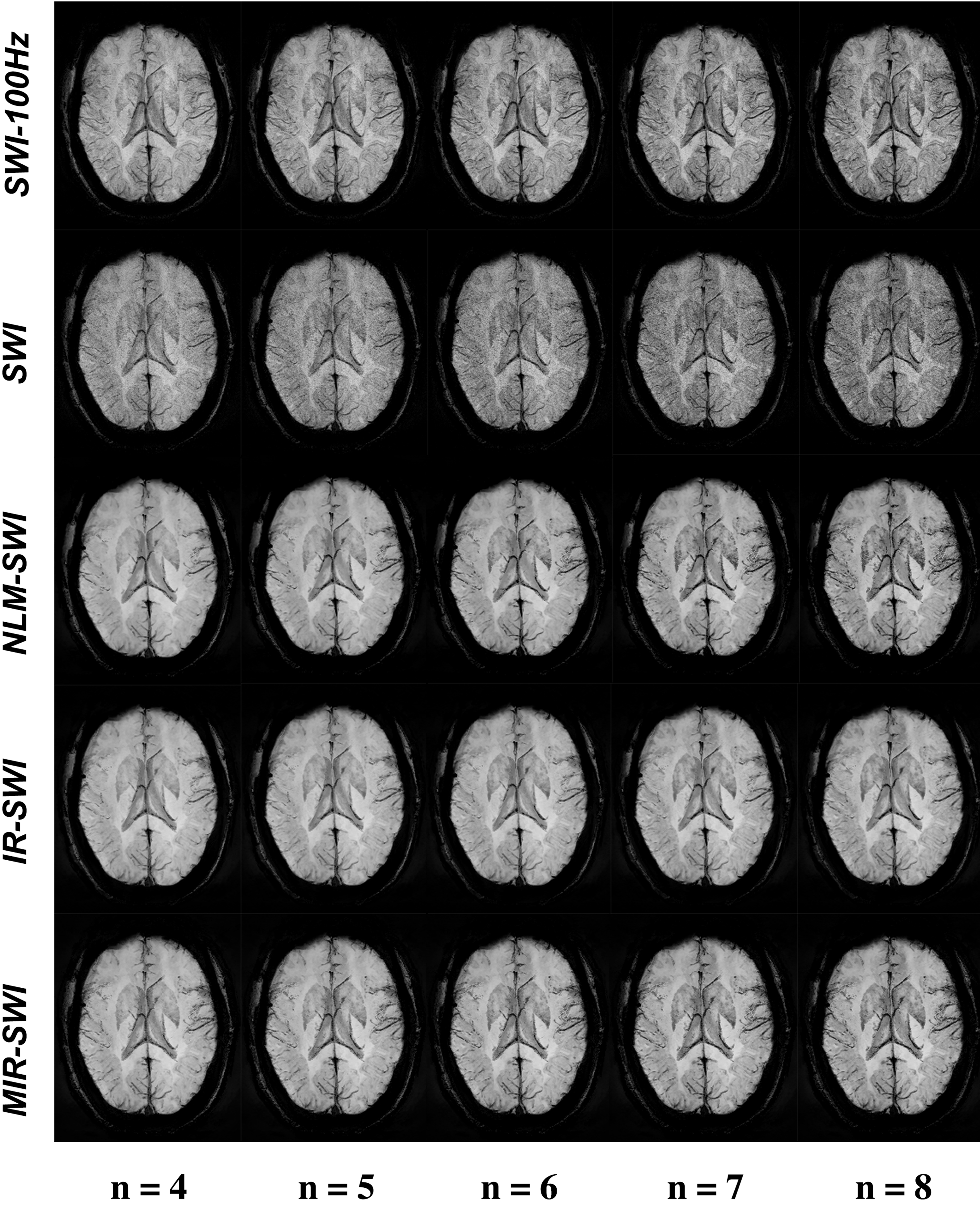

Supplement: S1 Fig — mIPs of the same targeted volume of 20 mm at varying n values. Subject #2. (TIF) [file pone.0126835.s001.tif]

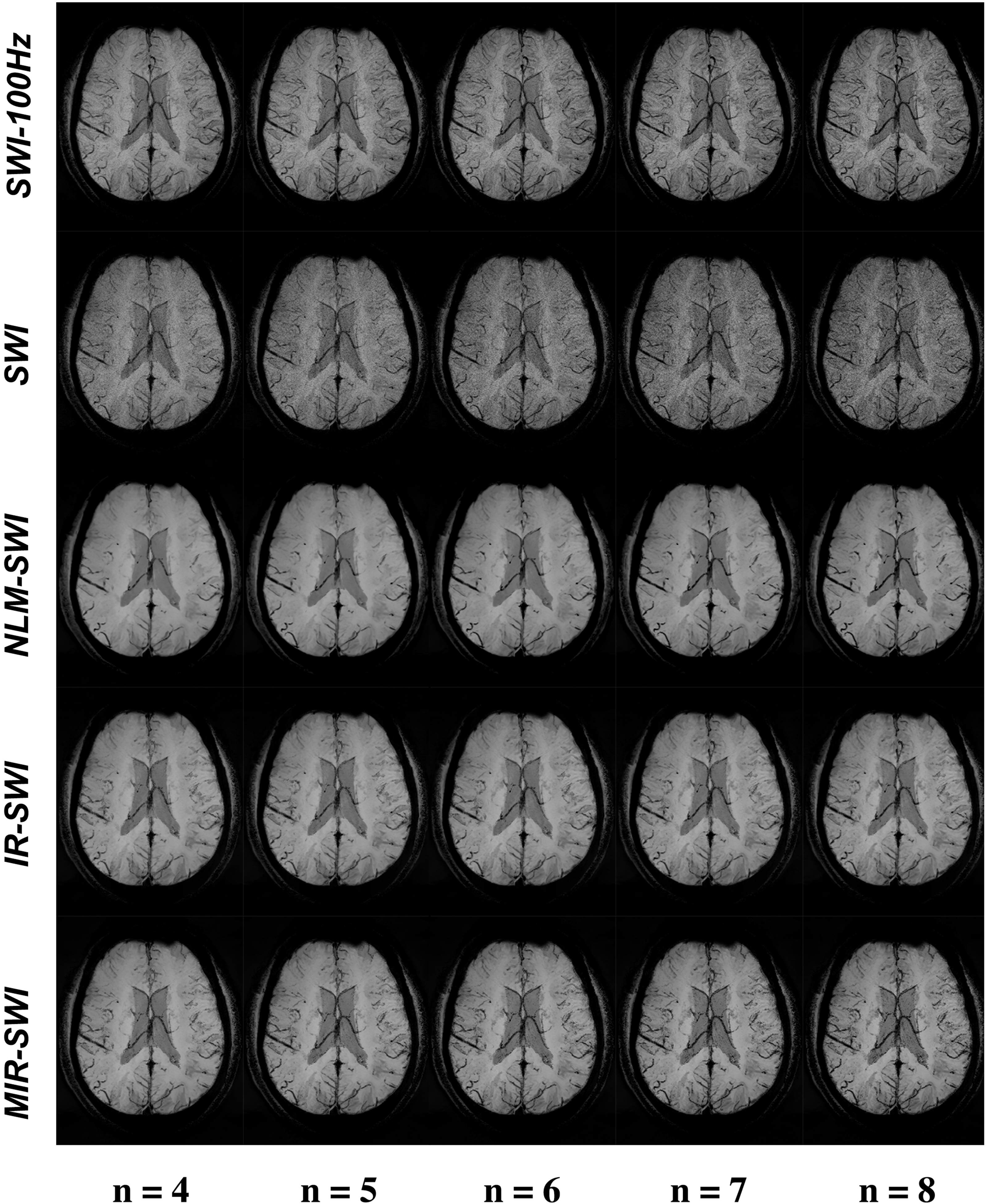

Supplement: S2 Fig — mIPs of the same targeted volume of 20 mm at varying n values. Subject #3. (TIF) [file pone.0126835.s002.tif]

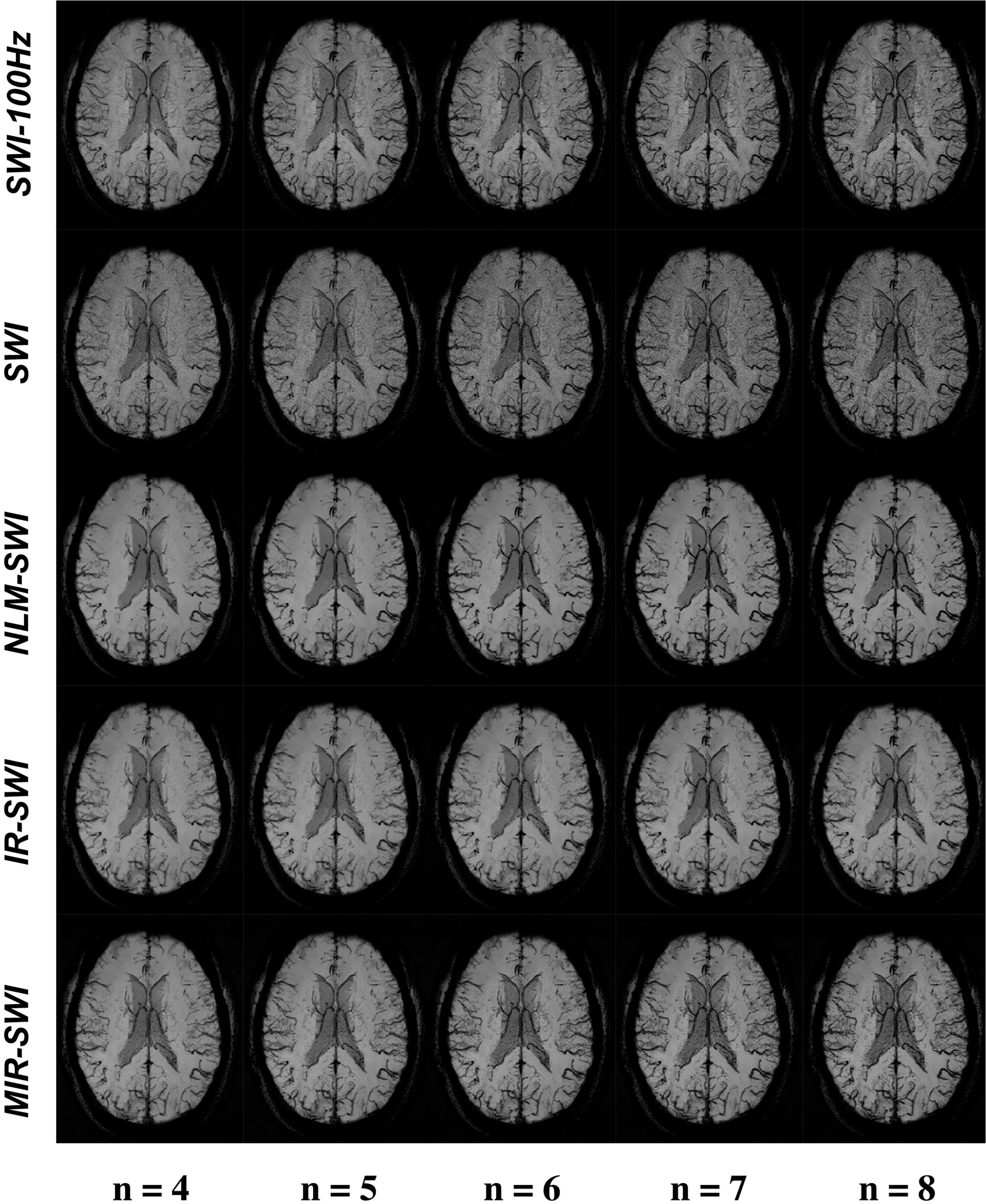

Supplement: S3 Fig — mIPs of the same targeted volume of 20 mm at varying n values. Subject #4. (TIF) [file pone.0126835.s003.tif]

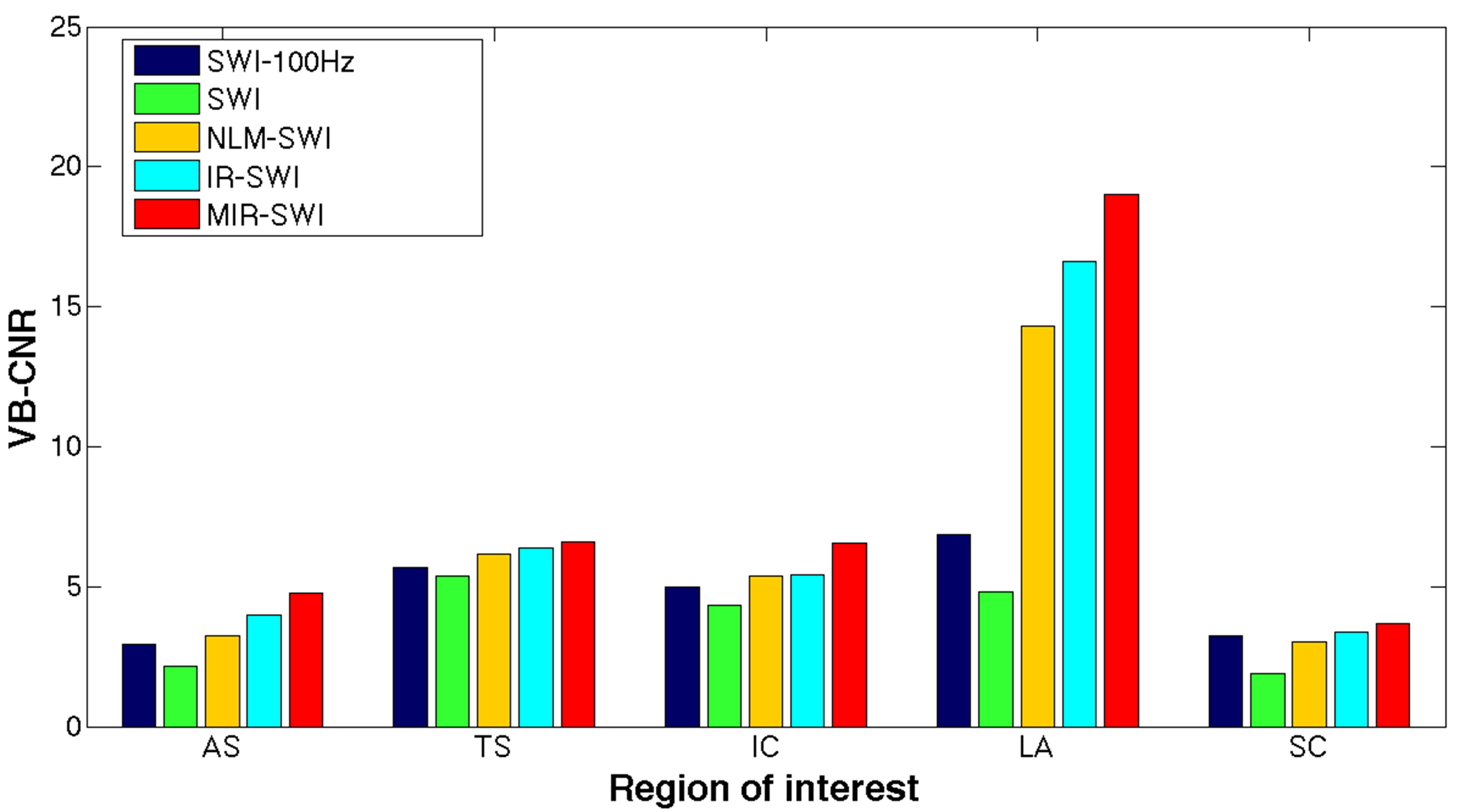

Supplement: S4 Fig — Same legend as Fig 4b. (TIF) [file pone.0126835.s004.tif]

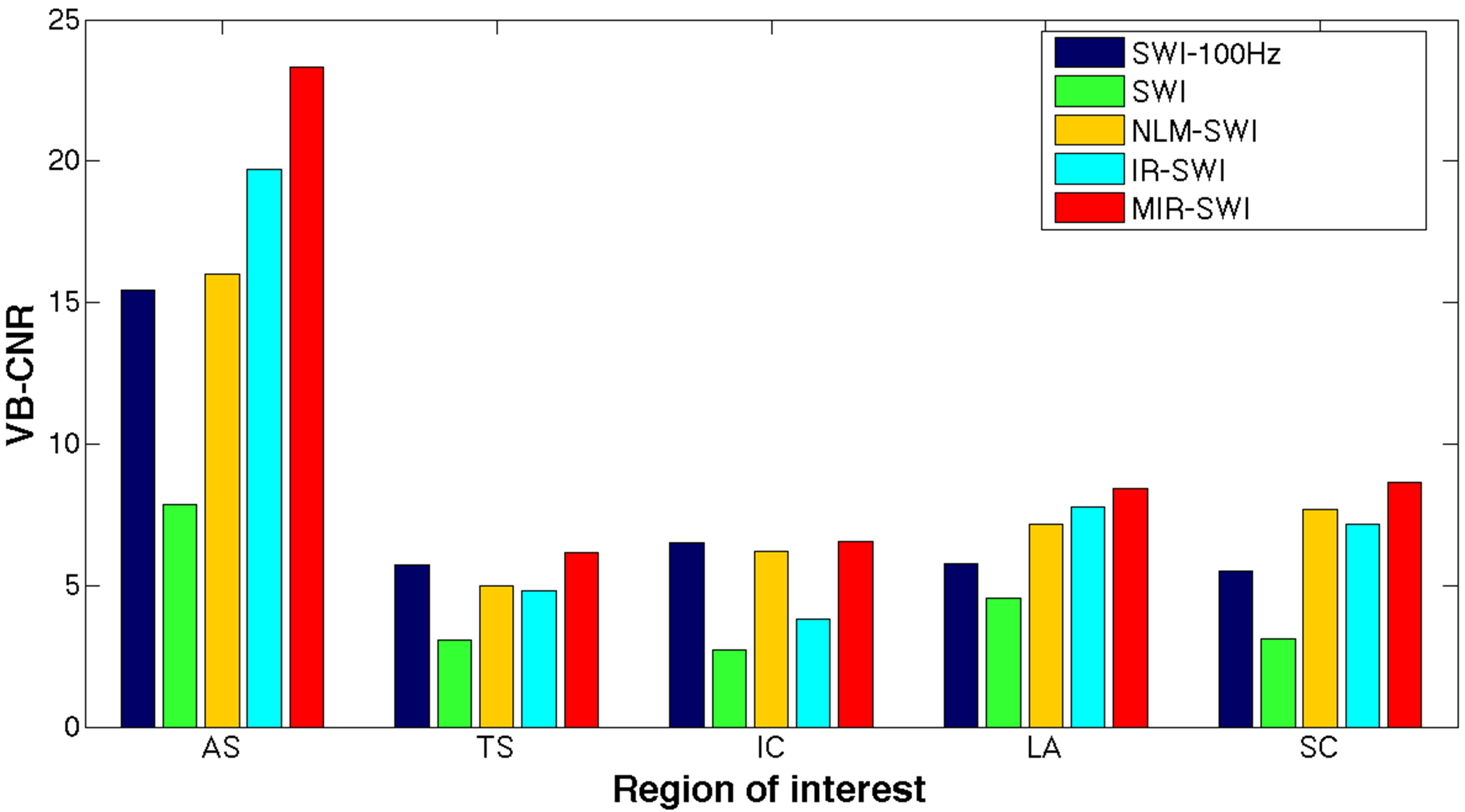

Supplement: S5 Fig — Same legend as Fig 4b. (TIF) [file pone.0126835.s005.tif]

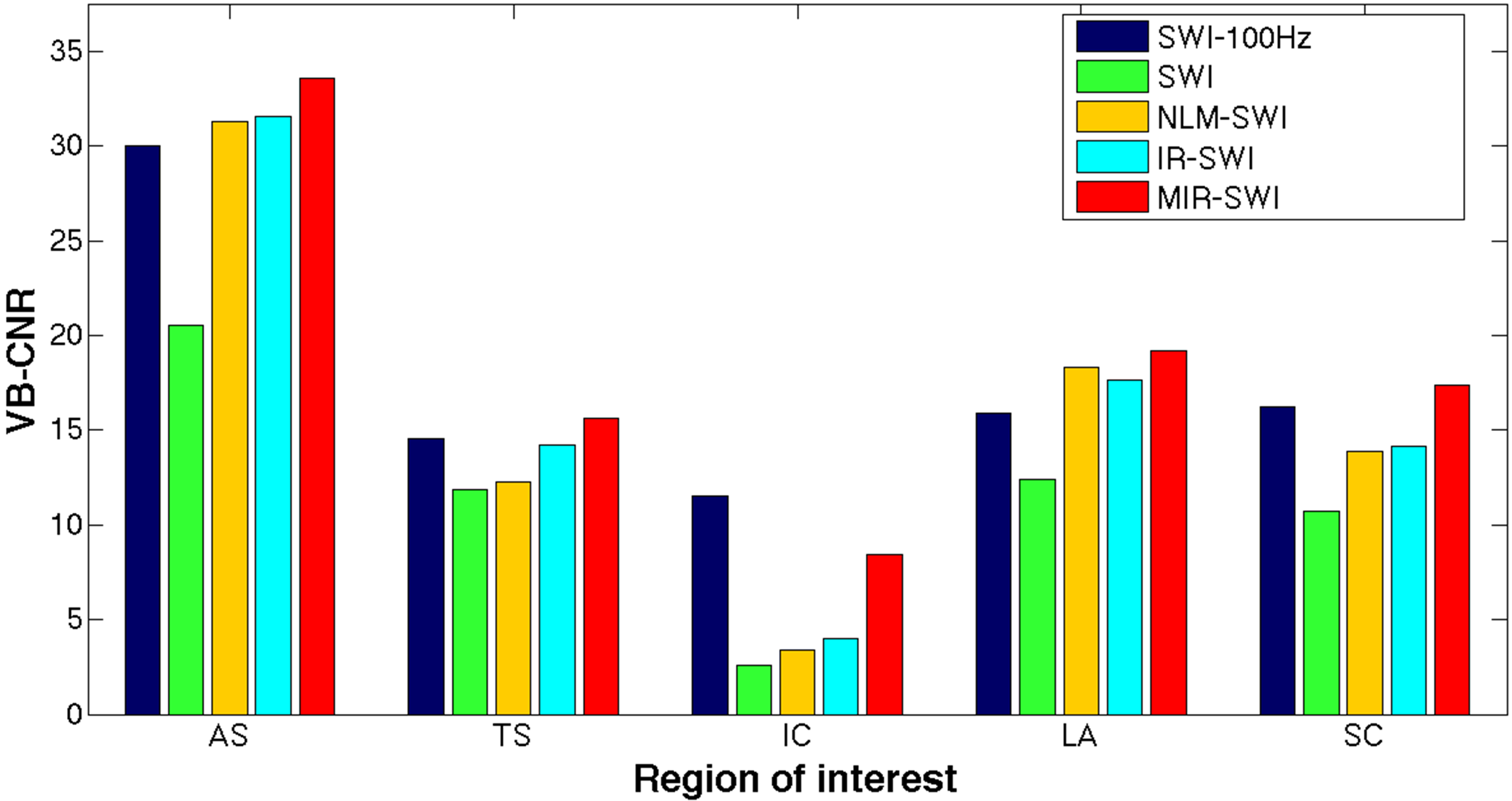

Supplement: S6 Fig — Same legend as Fig 4b. (TIF) [file pone.0126835.s006.tif]

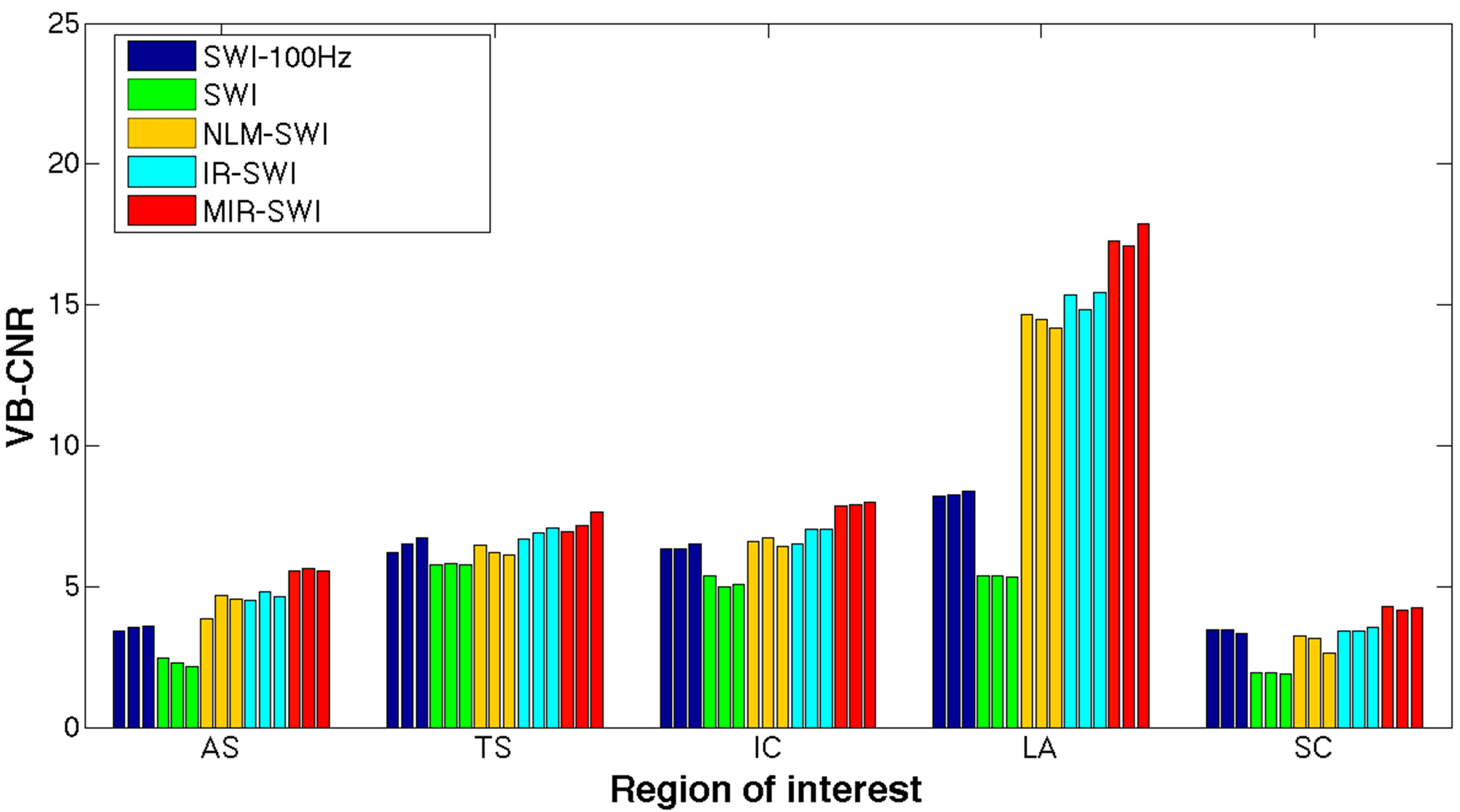

Supplement: S7 Fig — Same legend as Fig 7. (TIF) [file pone.0126835.s007.tif]

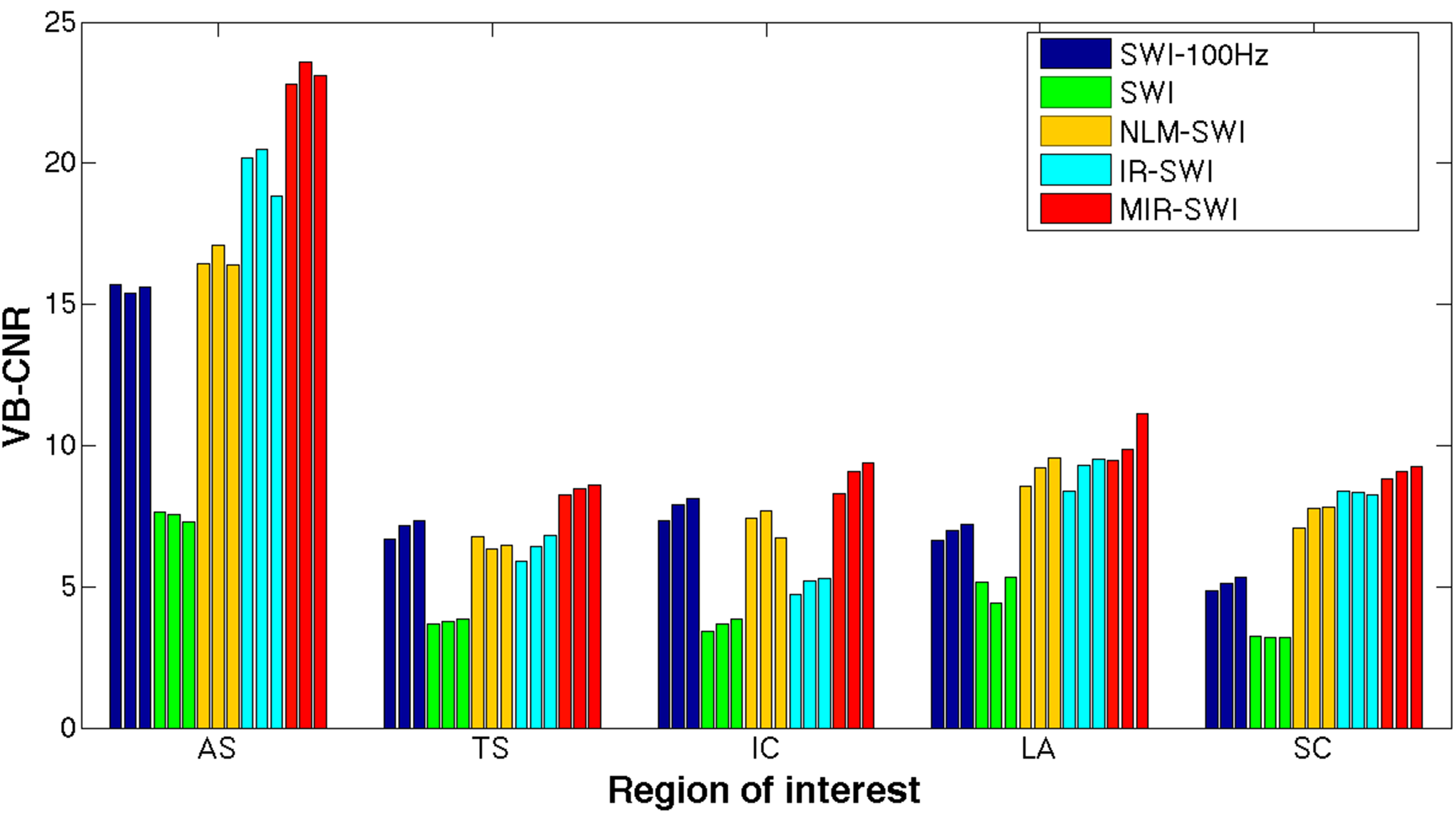

Supplement: S8 Fig — Same legend as Fig 7. (TIF) [file pone.0126835.s008.tif]

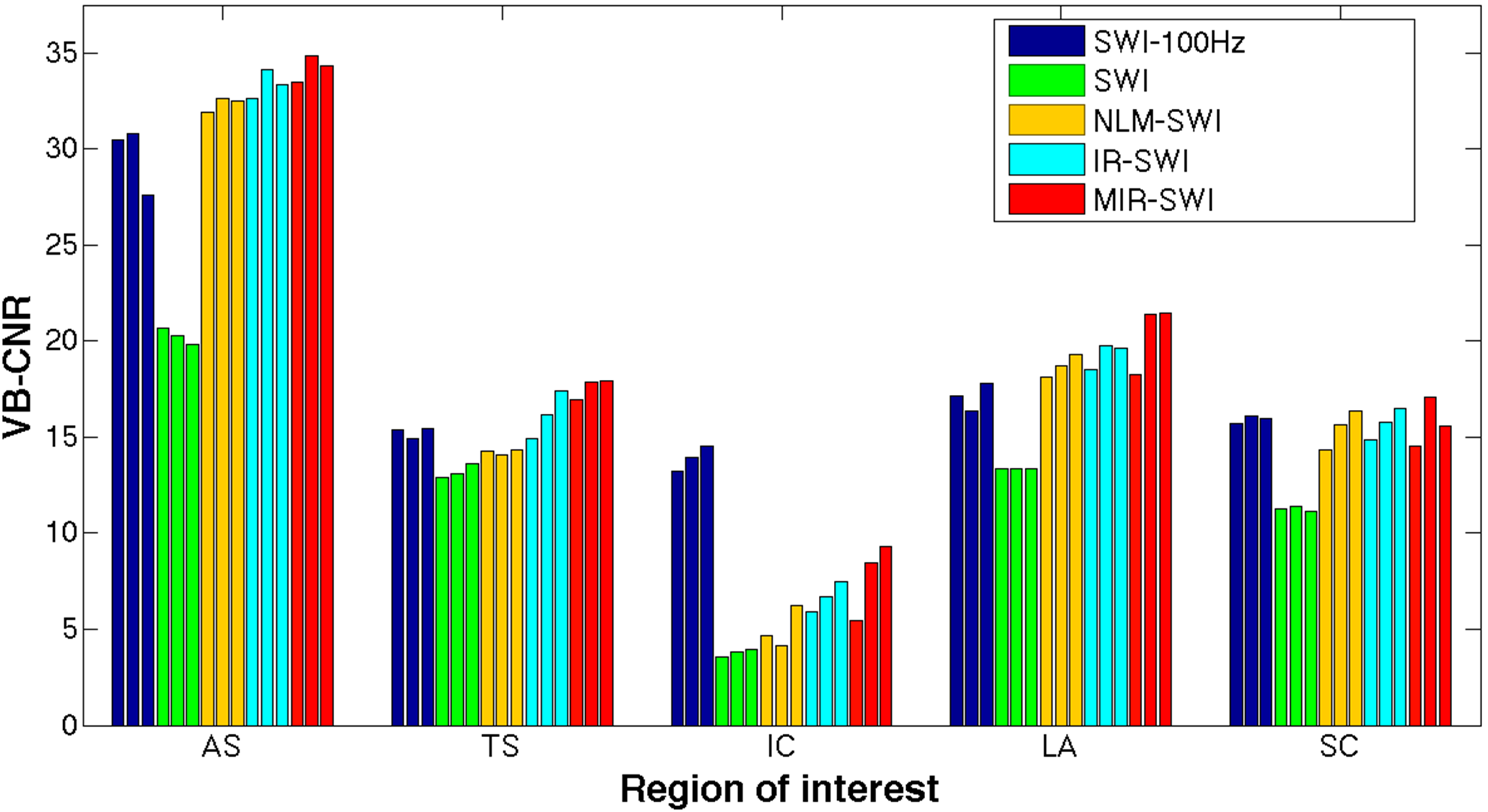

Supplement: S9 Fig — Same legend as Fig 7. (TIF) [file pone.0126835.s009.tif]
